# Quality of life improvement after a three-year course of sublingual immunotherapy in patients with house dust mite and grass pollen induced allergic rhinitis: results from real-life

**DOI:** 10.1186/s12955-017-0764-z

**Published:** 2017-09-29

**Authors:** Silviya Mihaylova Novakova, Maria Toncheva Staevska, Plamena Ivanova Novakova, Manuela Dimitrova Yoncheva, Maria Stoykova Bratoycheva, Nina Mihaylova Musurlieva, Valeri Dimitrov Tzekov, Dimitar Georgiev Nicolov

**Affiliations:** 1 Allergy Unit of Internal Consulting Department, University hospital St. George, “Peshtersko shosse” 66, Plovdiv, Bulgaria; 2 Clinic of Allergy and Asthma, Sofia Medical University, “Georgi Sofiiski”1, Sofia, Bulgaria; 3 Internal Consulting Department, University hospital “St. George”, “Peshtersko shosse” 66, Plovdiv, Bulgaria; 4 Faculty of Public Health, Department of Social Medicine and Public Health, Medical University-Plovdiv, “Vasil Aprilov”15A, Plovdiv, Bulgaria; 5 Medical Faculty, The Second Internal Department, Medical University-Plovdiv, “Vassil Aprilov”15A, Plovdiv, Bulgaria

**Keywords:** Allergic rhinitis, Quality of life, Sublingual immunotherapy, House dust mite, Grass pollen, Real life, Sensitization

## Abstract

**Background:**

Along with its high prevalence, the burden of allergic rhinitis rests upon the serious impact on quality of life of patients. Allergic rhinitis is associated with impairments in daily activities, work and school performance, and practical problems. Patients suffer from sleep disorders and emotional problems. Тhe advantages of sublingual immunotherapy on quality of life have only recently begun to emerge. The objective of this prospective real-life study was to evaluate the effect of a three-year course of sublingual immunotherapy with house dust mite (HDM) and grass pollen extracts on quality of life in adults with allergic rhinitis.

**Methods:**

A total number of 191 adult patients [105 (54,979%) men; mean age 27.3 years (SD-6.14)] with moderate to severe allergic rhinitis and clinically relevant sensitization to house dust mites or grass pollen were prospectively evaluated in the course of management of their disease. Health-related quality of life was assessed by Rhinoconjunctivitis Quality of Life Questionnaire at baseline and after three-year course of sublingual immunotherapy.

**Results:**

The mean overall Qol score assessed at baseline and at the end of the third year of treatment decreased significantly in patients treated with HDM extract (from 2.95 to 0.76) as well as with Grass pollen extract (from 2.83 to 1.22) (р < 0.001). The improvements in treated with HDM extract were as followed: activities – 3.52 to 0.68; sleep- 2.48 to 0.31; general problems – 1.79 to 0.49; practical problems – 3.57 to 0.68; nasal symptoms – 3.91 to 0.74; eye symptoms – 2.92 to 0.39; emotions – 3.03 to 0.39. The improvements in grass pollen group were: activities – 3.68 to 1.69; sleep- 1.85 to 0.84; general problems – 1.74 to 0.97; practical problems – 3.52 to 1.37; nasal symptoms – 3.72 to 1.57; eye symptoms – 3.58 to 1.3; emotions – 2.48 to 1.19.

**Conclusion:**

Our study conducted in real life provided evidence that a three-year course of SLIT with HDM extract as well as with grass pollen extract significantly increased QoL in patients with allergic rhinitis.

## Background

Allergic rhinitis is the most common allergic disease, affecting more than one third of the population worldwide [1]. Along with its high prevalence, the burden of the disease rests upon the serious impact on quality of life of patients. Symptoms of allergic rhinitis are exceptionally irritating and can significantly impact social functioning. Allergic rhinitis is associated with impairments in daily activities, work and school performance, and practical problems. Additionally, patients suffer from sleep disorder and emotional problems [2, 3]. Understandably, assessment of the severity of allergic rhinitis (AR) is based not on the severity of symptoms but on the impact of symptoms on the quality of life [4].

AR is a symptomatic disorder of the nose, induced after allergen exposure by Ig-E mediated inflammation of nasal membrane. It is often accompanied by allergic conjunctivitis and represents one common disease: allergic rhinoconjunctivitis [5]. AR and asthma often coexist and comprise the disorder of the united airway. The most common allergens that cause the disease are grass pollen, and house dust mite (HDM) [4].

Sublingual immunotherapy (SLIT) is a comparatively new form of allergen immunotherapy recommended by Allergic Rhinitis and its Impact on Asthma (ARIA) guidelines for adults with moderate to severe AR, sensitized to HDM and grass pollen [6, 7]. Recommended duration of treatment is three years [6]. As a long-lasting and self-administered treatment, it depends to a great extend on patients themselves and assessment of effectiveness in real life is essential. Furthermore, assessment of health-related quality of life (HRQoL) is recommended by the European Academy of Allergy and Clinical Immunology (EAACI) to be one of the clinical outcomes in allergen immunotherapy trials for allergic rhinoconjunctivitis [8].

In contrast to the standard, but more challenging for application subcutaneous immunotherapy, the advantages of SLIT on quality of life have only recently begun to emerge. Significantly fewer investigations have explored the impact of SLIT on quality of life [9]. Results from several randomized double-blind placebo control (RDBPC) studies on efficacy of pollen SLIT on health-related quality of life (HRQoL) in patients with AR have been published, but the data from real life studies are scarce [10–13]. Data from RDBPC studies on efficacy of HDM SLIT are limited with assessment on the first year of treatment only [14, 15]. To the best of our knowledge there are no publications on the impact of HDM SLIT on HRQoL in real life after the completion of the recommended duration of treatment.

### Objective

The objective of this prospective real-life study was to evaluate the effect of a three-year course of SLIT with HDM and grass pollen extracts on quality of life in adults with AR.

## Methods

### Study design

This study was conducted in the allergy unit of the University hospital St. Georgе - Plovdiv, Bulgaria. All patients were referred for treatment either by their general practitioners or were self-referred. The study was designed to include adults, eligible for SLIT, who completed a three-year course of HDM SLIT and grass pollen SLIT as a routine management according to ARIA guideline. It was approved by the review board of the University Hospital and performed according to declaration of Helsinki. Informed consent was obtained from all participants. The patients were evaluated before initiation of immunotherapy and at the end of the third year of treatment. In between, follow up was performed over the course of treatment as usual. Physical examination and assessment of symptom severity and control were performed. Each patient was evaluated by the same physician. Assessment was performed throughout the year for house-dust mites HDM SLIT and in May and June – months with the highest grass pollen concentration for pollen SLIT.

### Patients

Patients with AR with evidence of clinically relevant sensitization and symptoms, not-well controlled with pharmacotherapy were included according to physician^’^s judgment.

Diagnosis of AR was made on the basis of detailed clinical history, a complete physical examination and positive skin prick test in conformity with the validated criteria [5]. Sensitization was determined by skin prick-test, according to EAACI guidelines [16].

Health-related quality of life (HRQoL) was assessed by interviewer-administered Bulgarian version of Rhinoconjunctivitis Quality of Life Questionnaire (RQLQ) [17]. This disease-specific questionnaire is designed for adults and consists of 28 items, distributed in 7 domains: activities – three items; sleep – three items; general problems – seven items; practical problems – three items; nasal symptoms – four items; eye symptoms – four items; emotions – four items. Patients are scored on a 7-point scale, from 0 to 6. Lower scores indicate better QoL. An average change in score of 0.5 pеr item and for overall QoL is the minimal clinically important difference [18]. Patients were interviewed by the physician at baseline and on the third year upon the completion of the recommended SLIT duration.

Duration and severity of AR were assessed and patients were classified according to ARIA classification [5].

Control was assessed by Bulgarian version of Rhinitis Control Assessment Test (RCAT) [19].

SLIT was conducted with standard extract of HDM or a mixture of pollen extracts of five grasses and four cereals [Staloral® 300 IR (Stallergens, France)] as sublingual drops, according to the schedules, recommended by the manufacturer. The treatment was administered perennially in patients with HDM allergy and pre- and co-seasonal in allergic to grass pollen. All adverse reactions were recorded.

#### Statistical analysis

The distribution of continuous samples was assessed by One–Sample Kolmogorov-Smirnov Test. For comparison, independent and paired samples t-test was used for quantitative data and Fisher’s exact test for qualitative data. *P*-value <0.05 was considered as statistically significant. Data were analyzed using IBM SPSS Statistics-20 (Chicago, IL, USA).

## Results

A total number of 191 adult patients [105 (54.97%) male; mean age 27.3 years (SD-6.14)] with moderate to severe AR and clinically relevant sensitization to HDM or grass pollen were prospectively evaluated in the course of management of their disease. HDM SLIT was performed on 76 (39.8%) patients and grass pollen SLIT – on 115 (60.2%). Age, gender, severity of AR, in relation to type of sensitization and concomitant diseases are presented in Table 1.

**Table 1:** Patients’ characteristics

Characteristics	Type of sensitization	
	HDM (*n* = 76)	Grass pollen (*n* = 115)
Age (in years)		
mean (SD)	26,10 (5,85)	25,73 (6,43)
Range	18–48	18–46
Gender:		
Male	42 (55.26%)	63 (54.78%)
Female	34 (44.74%)	52 (45.22%)
Type of AR:		
moderate/severe intermittent	0	31 (26.96%)
moderate/severe persistent	76 (100%)	84 (73.04%)
Concomitant diseases:		
asthma:	28(36.84%)	41(35.65%)
atopic dermatitis	5(6.58%)	7(6.09)

*n* number, *AR* allergic rhinitis, *HDM* house dust mite

No significant difference in age and gender between two groups was established. All patients were with moderate to severe AR and those with persistent AR predominated.

**Fig. 1:**
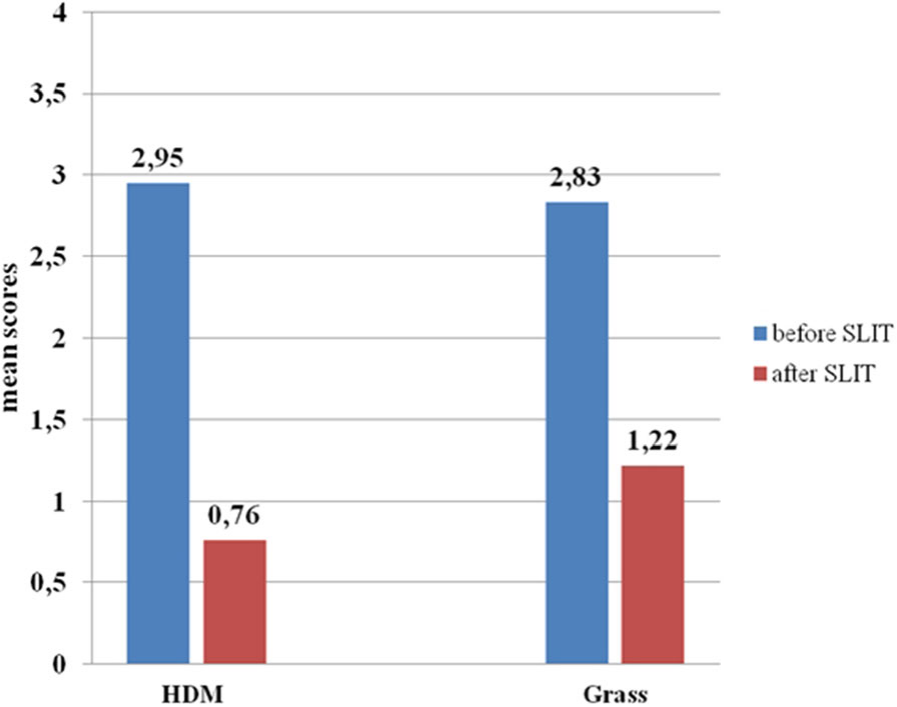
Overall quality of life before and after house dust mite (HDM) and grass pollen sublingual immunotherapy (SLIT) assessed by Rhinoconjunctivttis Quality of Life Questionnaire. The scores are shown as adjustive means with 95% confidence interval – *p* < 0.001

The mean overall Qol score assessed at baseline and at the end of the third year of treatment decreased significantly in patients treated with HDM extract (from 2.95 to 0.76) as well as with Grass pollen extract (from 2.83 to 1.22) (*р* < 0.001). The effectiveness data representing the differences in overall QoL with comparison between HDM and Grass SLIT are presented in Fig. 1.

**Fig. 2:**
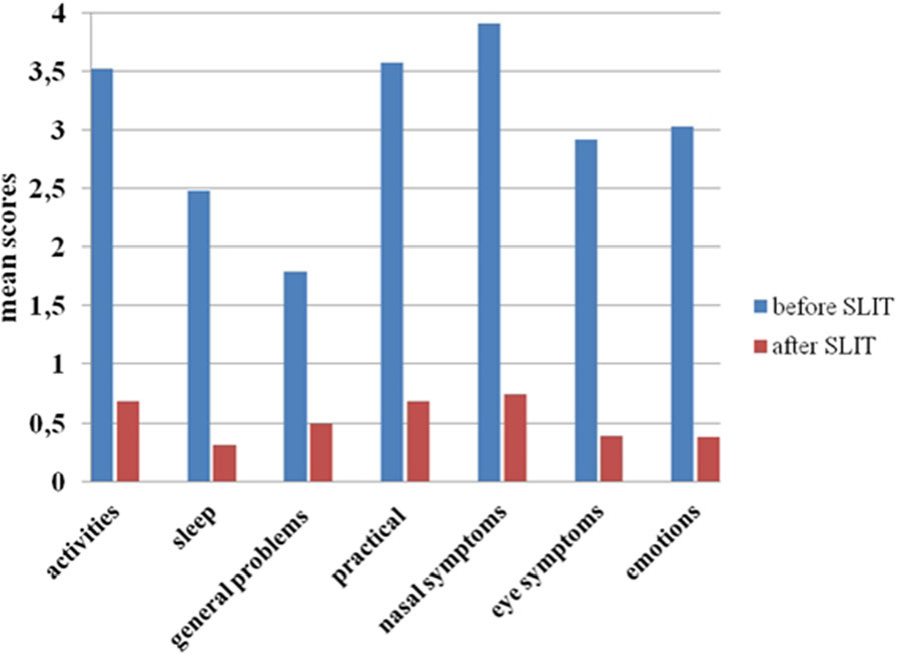
Changes in individual domains of Rhinoconjunctivttis Quality of Life Questionnaire in treated with house dust mite sublingual immunotherapy (SLIT). The scores are shown as adjustive means with 95% confidence interval - *p* < 0.001

**Fig. 3:**
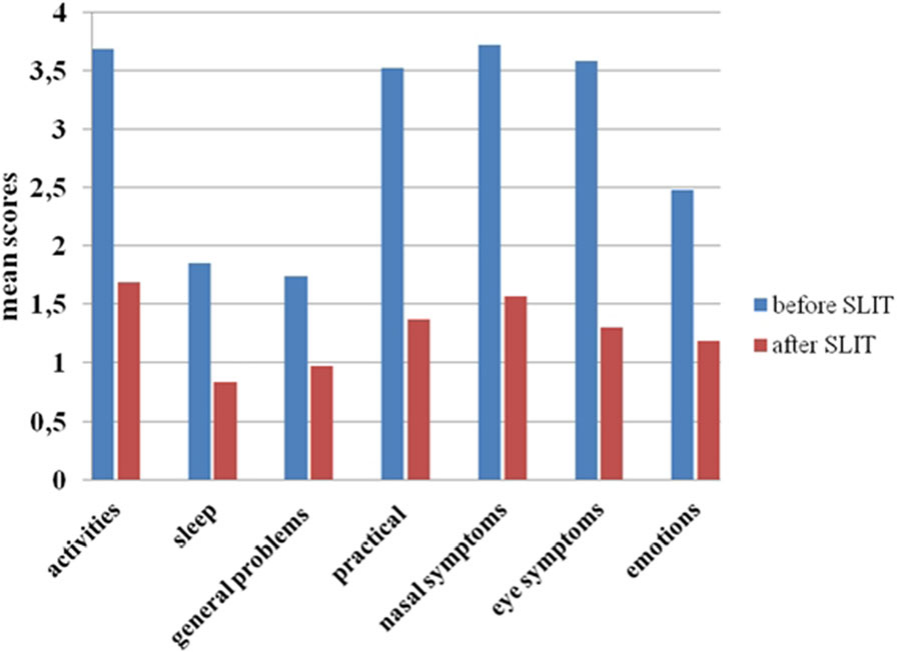
Changes in individual domains of Rhinoconjunctivttis Quality of Life Questionnaire in treated with grass pollen sublingual immunotherapy (SLIT). The scores are shown as adjustive means with 95% confidence interval - *p* < 0.001

When comparing both treatment no significant difference was found (*t* = 0.45) (*p* > 0.1). For the individual domains of RQLQ the mean values of scores were analyzed. The results are presented in Figs. 2 and 3.

The differences in each domain in both groups of sensitization are presented in Table 2 and Table 3 in details.

**Table 2:** Comparison between quality of life scores before and after sublingual immunotherapy with house dust mites extract assessed by RQLQ

Domains			mean (SD)	Difference	*P*
1.	Activities	before	3.52 (1.41)	2.84	< 0,001
		after	0.68 (0.93)		
2.	Sleep	before	2.48 (1.8)	2.17	< 0,001
		after	0.31 (0.58)		
3.	General problems	before	1.79 (1.55)	1.3	< 0.001
		after	0.49 (0.77)		
4.	Practical problems	before	3.57 (1.68)	2.89	< 0.001
		after	0.68 (1.01)		
5.	Nasal symptoms	before	3.91 (1.66)	3.17	< 0.001
		after	0.74 (0.99)		
6.	Eye symptoms	before	2.92 (2.01)	2.53	< 0.001
		after	0.39 (0.74)		
7.	Emotions	before	3.03 (1.43)	2.65	< 0.001
		after	0.38 (0.69)		
Overall QoL		before	2.95 (1.32)	2.19	<0.001
		after	0.76 (0.55)

**Table 3:** Comparison between quality of life scores before and after sublingual immunotherapy with Grass pollen extract assessed by RQLQ

Domains			mean (SD)	Difference	*P*
1.	Activities	before	3.68 (1.39)	1.99	< 0.001
		after	1.69 (1.7)		
2.	Sleep	before	1.85 (1.77)	1.01	< 0.001
		after	0.84 (1.46)		
3.	General problems	before	1.74 (1.48)	0.77	< 0.001
		after	0.97 (1.26)		
4.	Practical problems	before	3.52 (1.54)	2.15	< 0.001
		after	1.37 (1.39)		
5.	Nasal symptoms	before	3.72 (1.36)	2.15	< 0.001
		after	1.57 (1.4)		
6.	Eye symptoms	before	3.58 (1.72)	2.28	< 0.001
		after	1.30 (1.33)		
7.	Emotions	before	2.48 (1.58)	1.29	< 0.001
		after	1.19 (1.14)		
Overall QoL		before	2.83	1.61	<0.001
		after	1.22		

Among patients treated with HDM extract, the greatest difference before and after SLIT was observed for “Nasal symptoms” – 3.17 (*p* < 0.001), followed by “Practical problems” - 2.98, (*p* < 0.01) and “Activities” - 2.84 (*p* < 0.01) domains. In the group of patients, treated with Grass pollen extract, the greatest difference was determined in “Eye symptoms” - 2.28 (*p* < 0.01), followed by “Nasal symptoms” and “Practical problems” - 2.14 (*p* < 0.01) domains.

No serious adverse reactions were recorded. In the HDM extract treated group 22 patients (28.95%) expressed local oral and 5 (6.58%) gastrointestinal reactions. In the Grass pollen extract treated group 33 patients (28.70%) expressed local oral and 7 (6.09%) gastrointestinal reactions.

## Discussion

It is now recognized that AR comprises more than the classical symptoms of sneezing, rhinorrhoea and nasal congestion. AR affects multiple parameters including, physical, psychological and social functioning. It has a serious impact on sleep and productivity at work/school [20, 21]. AR is associated with impaired HRQoL and presents an important aspect to consider in managing patients.

Several validated tools for assessing HRQoL in rhinitis are currently available [22]. The most frequently used rhinoconjunctivitis specific instrument, utilized in allergen immunotherapy trials is RQLQ, indicating the reason the Bulgarian version of the questionnaire was chosen for our study with the permission from the author. Following the instructions of ARIA guidelines, all patients included in our study were adults with moderate to severe AR.

Recommended adequate duration of SLIT is three years, indication the chosen period for the assessment in our study. It was a challenge for us to maintain follow up with patients in real life for such an extended. Additionally, by our knowledge there are no publications on assessment in real life for such a long period corresponding to recommended duration of treatment.

A significant positive impact on quality of life was found in patients treated with HDM extract. The result was comparable to what was reported in two DBPCT conducted for one year [14, 15]. The improvement of quality of life in patients treated with Grass pollen extract was established as well. Nelson et al. demonstrated that Timothy grass SLIT improved QOL after one season of treatment in a randomized study [11]. In another DBPC trial with grass pollen tablets the authors concluded that this treatment improved QOL with sustained efficacy two years after treatment completion [13]. Although not entirely novel the observations in our study have important clinical relevance since it is well established that results from clinical trials are not always repeated in real life. Adherence to SLIT in real-life, especially to the recommended prolonged course could be a problem and compromises the efficacy, demonstrated in clinical trials. In our study, we established that SLIT is effective in improvement Qol in real life.

A direct comparison of Qol in patients, sensitized to HDM and Grass pollen is difficult. Patients have different perception of perennial symptoms in HDM sensitization and seasonal symptoms in Grass pollen sensitization. However, when comparing relative differences of overall Qol scores in the presented study no significant difference was established. This observation confirmed that both HDM and Grass pollen SLIT can improve QoL in patients with AR.

In the group of patients, treated with HDM SLIT significant reduction in all domains was observed, including “nasal symptoms”, “practical problems” and “activities”. These findings are important from clinical point of view. Nasal symptoms contribute significantly to impaired HRQoL. Nasal congestion is often associated with sleep disturbances leading to daytime fatigue and somnolence, and decreased cognitive functioning. Nasal symptoms are a result of allergic inflammation. There is evidence that QoL in AR is strictly associated with allergic inflammation [23]. We explain the observed reduction in nasal symptom with immunological mechanism of action of SLIT and its direct influence on allergic inflammation and indirect - on HRQoL. As a consequence of their symptoms patients with AR are forced to carry handkerchiefs or tissues, and need to rub and blow their noses repeatedly. This could potentially interfere with their social interaction, activity limitation and social isolation. Improvement of “practical problems” contributed to overall improvement of HRQoL as a result of SLIT. It was already reported that more than 80% of patients with moderate to severe allergic rhinitis had reduction in their daily activities [24]. A significant improvement in “activities” items was established on the third year of treatment.

A high score of the domain “emotions” was noted in the group of patients sensitized to HDM before SLIT. It is known that AR may contribute to patients report of depressed and/or anxious mood. Significant improvement in the emotions of patients after three years of SLIT was demonstrated. The impact of SLIT on all other aspects that affect HRQoL might explain the improvement of their emotional wellbeing. All of these observations provided additional evidence that while there continue to be some debates on its clinical efficacy [25], our study clearly demonstrated that HDM SLIT was effective treatment with respect to HRQoL.

We observed a significant decrease in all domains in the group of patients treated with Grass pollen extract group of patients. The greatest difference in “eye symptoms” was demonstrated followed by “nasal symptoms” and “practical problems”. Eye symptom is the aspect that, together with nasal symptoms, congestion in particular, has been found to strongly affect HRQoL [23, 26]. Eye symptoms have a significant impact on daily activities and work or school performance. At the same time they are some of the most difficult to control. Eye symptoms are related to one of the AR phenotypes - SCUAD (Severe Chronic Upper Airway Diseases) which is a serious therapeutic challenge. The results from our study demonstrated a high score for “eye symptoms” domaim before initiation of SLIT, especially in the group of patients, treated for grass pollen allergy. Relevant improvement was achieved after three years of SLIT.

The only real life study on SLIT to compare our results with was published in 2010 [27]. This study demonstrated significant reduction in all assessed domains, clinically relevant for activities, practical problems, and nose and ocular symptoms after one year of SLIT in polysensitized patients. We established significant decrease in the same domains on the third year of SLIT, providing conformation of sustained and stable effectiveness of SLIT in real life.

As expected, the prevalence of adverse reactions was low and in conformity with already published data [28, 29]. We confirm that SLIT is safe and well tolerated by patients.

There are some limitations to the study. Our results refer to a specific product and there was no control group. Another possible limitation is that concomitant diseases can affect HRQoL, thus hindering the results. However, the questionnaire used for assessment is a disease-specific. The main strength of the study is that data collection was performed in routine practice setting and obtained from a large number of patients who completed three-year course of SLIT according to the recommendation for this treatment. Moreover, the outcome which was assessed was in accordance with the recent recommendations for standardized clinical outcomes used in allergen immunotherapy trials for AR. We believe this reinforces the value of our findings.

## Conclusions

AR has a significant impact on QoL and its assessment is an important tool in management of the disease. Our study conducted in real life provides evidence that a three-year course of SLIT with HDM extract as well as with grass pollen extract significantly increases QoL in patients with AR.

## Abbreviations

AR: Allergic rhinitis
ARIA: Allergic Rhinitis and Impact on Asthma
EAACI: European Academy of Allergy and Clinical Immunology
HDM: House dust mite
HRQoL: Health related quality of life
QoL: Quality of life
RDBPC: Randomized double-blind placebo control
RQLQ: Rhinoconjunctivitis Quality of Life Questionnaire
SLIT: Sublingual immunotherapy
